# Magnitude and pattern of Arctic warming governed by the seasonality of radiative forcing

**DOI:** 10.1038/srep38287

**Published:** 2016-12-02

**Authors:** R. Bintanja, F. Krikken

**Affiliations:** 1 Royal Netherlands Meteorological Institute (KNMI), Utrechtseweg 297, 3731 GH De Bilt, The Netherlands; 2 Meteorology and Air Quality Section, Wageningen University, Wageningen, The Netherlands

## Abstract

Observed and projected climate warming is strongest in the Arctic regions, peaking in autumn/winter. Attempts to explain this feature have focused primarily on identifying the associated climate feedbacks, particularly the ice-albedo and lapse-rate feedbacks. Here we use a state-of-the-art global climate model in idealized seasonal forcing simulations to show that Arctic warming (especially in winter) and sea ice decline are particularly sensitive to radiative forcing in spring, during which the energy is effectively ‘absorbed’ by the ocean (through sea ice melt and ocean warming, amplified by the ice-albedo feedback) and consequently released to the lower atmosphere in autumn and winter, mainly along the sea ice periphery. In contrast, winter radiative forcing causes a more uniform response centered over the Arctic Ocean. This finding suggests that intermodel differences in simulated Arctic (winter) warming can to a considerable degree be attributed to model uncertainties in Arctic radiative fluxes, which peak in summer.

The warming of the Arctic regions and the associated sea ice retreat are among the most prominent features of ongoing and projected climate change. A host of regional feedback mechanisms, mostly related to sea ice, contribute to Arctic warming being much stronger than the global response^1^,^2^,^3^,^4^,^5^,^6^,^7^,^8^. Simulating Arctic warming using climate models^9^,^10^ involves many uncertainties though, as indicated by the large intermodel differences. This may be attributed to uncertainties in the magnitude of climate feedbacks, to intermodel differences in the representation of important physics such as radiation^11^, and to uncertainties in the radiative forcing. In any case, the interrelation between Arctic warming and sea ice decline clearly is a vital issue^12^, since this connection involves many of the relevant regional Arctic feedbacks as well as the shortwave and longwave radiation characteristics that govern the Arctic climate response.

Over the 21^st^ century, the CMIP5 RCP8.5 scenario^13^ (see Methods) projects a model-mean annual mean Arctic warming of 8.5 ± 4.1 °C (Fig. 1a) accompanied by a 49 ± 18% retreat in sea ice cover, with summer sea ice having largely vanished in 2100. Arctic warming exhibits a very pronounced seasonal cycle, however, with exceptionally strong warming in the winter months (up to 14.1 ± 2.9 °C in December) and only moderate warming during the summer season (Fig. 1b). Associated with this surface-based warming is a strong increase in precipitation (up to 60%), which has been attributed mainly to reduced sea ice cover and the associated strong increase in evaporation from the open Arctic Ocean^14^. The projected precipitation changes therefore also peak in winter, meaning that not only the magnitude but also the seasonal imprint of Arctic warming has important ramifications for various components of the Arctic (climate) system^15^. Evidently, the changing seasonal cycle in the Arctic climate will have profound effects on Arctic ecosystems, emerging economic activities (e.g. shipping, fishery, mining, tourism) and may even impact the climate in other parts of the world^16^,^17^,^18^, for instance through changes in sea level and global ocean currents^14^,^15^. It is therefore imperative to identify and quantify the climate mechanisms and feedbacks that cause the huge seasonal range in Arctic warming.

Until now, Arctic warming and its seasonal variation have been addressed primarily by studying various climate feedbacks^1^,^2^,^3^,^4^,^5^,^7^,^19^, related to, among others, surface albedo, atmospheric humidity and clouds, and poleward transport of dry static and latent heat. An important aspect of the seasonal response is the ice-albedo feedback, which operates mainly in the spring/summer seasons. However, this feedback contributes to winter warming^20^ through interacting with storage (in summer) and release (in winter) of heat in the Arctic Ocean^2^,^5^,^21^. Arctic winter warming is further amplified by feedbacks that operate in wintertime, such as the lapse-rate feedback^7^. Another aspect that modulates the (seasonal) climate response in the Arctic is the direct radiative forcing by increasing concentrations of greenhouse gases, which does in fact exhibit a seasonal signature^22^.

## Results

**Figure 1:**
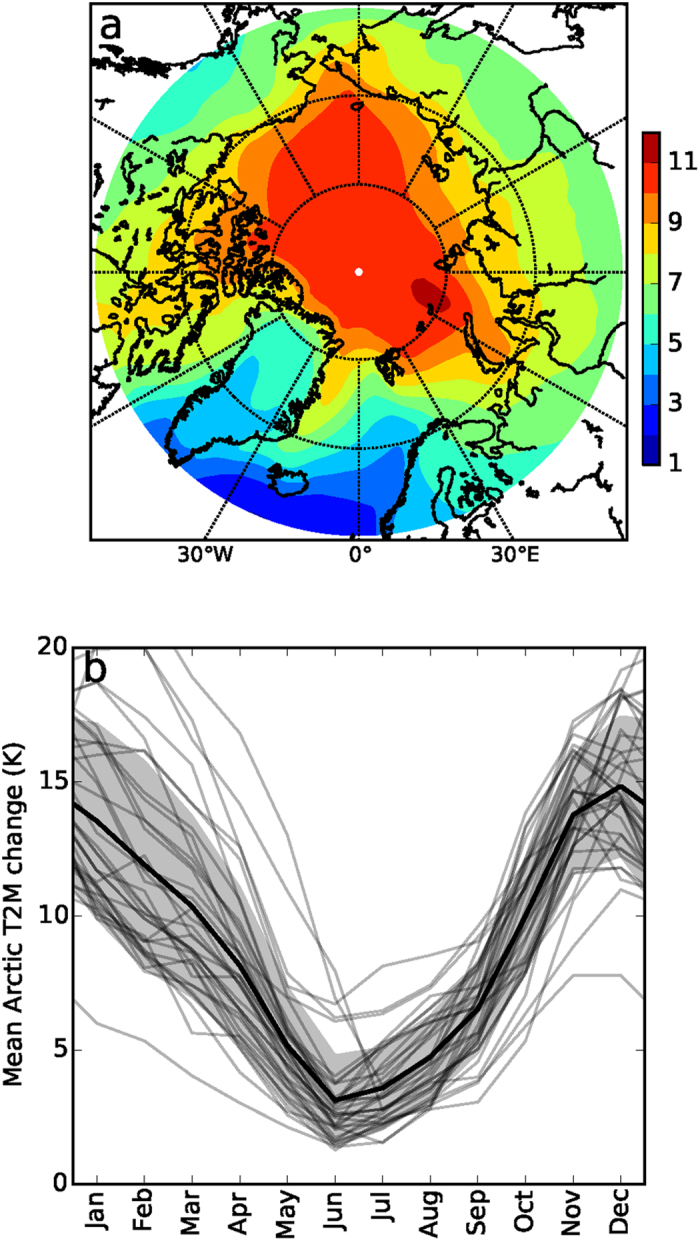
Projected 21^st^-century Arctic (70°–90°N) near-surface warming (T2m) using the Coupled Model Intercomparison Project, phase 5 (CMIP5) model ensemble. (**a**) Model-mean annual mean Arctic near-surface temperature change, (**b**) Annual cycle in Arctic near-surface warming, with the grey envelope representing the standard deviation of the intermodel mean. Dark-grey lines denote individual CMIP5 models. Results are for the strong (RCP8.5) forcing scenario, in which the combined greenhouse, aerosol and other radiative forcings in the year 2100 totals 8.5 W m^−2^ (ref. 13) relative to the pre-industrial climate. 21^st^-century trends in Arctic temperature are defined as the difference between the means over the periods 2091–2100 and 2006–2015 (37 models). The map in (**a**) was made using Python V2.7 (www.python.org).

**Figure 2:**
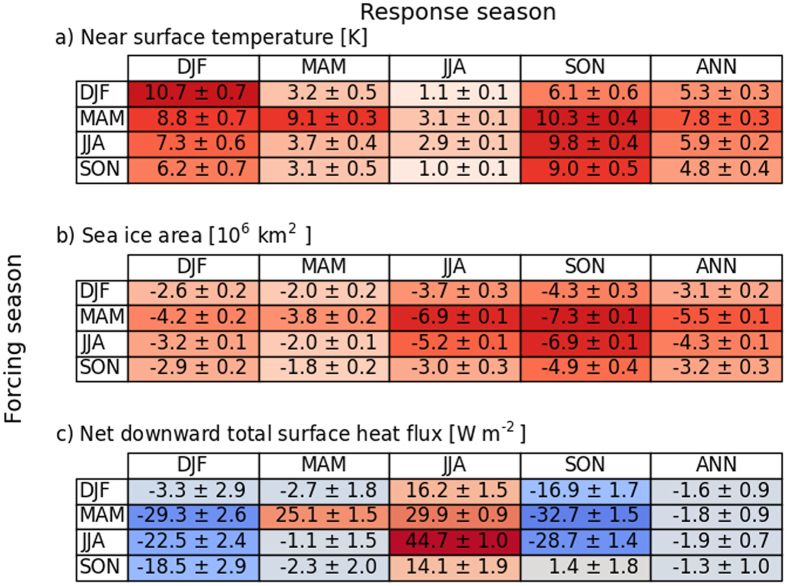
Simulated seasonal and annual response resulting from a surface radiative forcing in various seasons (see Methods). Surface air temperature (in K, upper panel), Sea ice area (in 10^6^ km^2^, middle panel), Net downward total surface heat flux (in W m^−2^, bottom panel), which is the total of the net shortwave and longwave radiative fluxes and the sensible and latent heat fluxes. Note that the 30 W m^−2^ additional downward forcing (see Methods) is included in the response value for the forcing season (as an example, the 44.7 W m^−2^ JJA response for JJA forcing includes the 30 W m^−2^ forcing). ANN represents the annual mean response. The uncertainties represent the 95% cadditional downward forcing (see Methods) is included in the response value for the forcing season (as an example, the 44.7 W m^−2^ JJA confidence interval of the mean, where seasonal (annual) means are used to evaluate the seasonal (annual) response uncertainties. Colouring indicates the magnitude and sign of the responses..

Here we use a state-of-the-art global climate model (EC-Earth^9^, see Methods) in idealized climate (‘ghost’) forcing simulations to quantify the effect of seasonality in radiative forcing on the magnitude and pattern of seasonal Arctic warming. To infer the climate response throughout the year resulting from forcings in different seasons, an artificial longwave radiative forcing was applied to the surface for each season separately (see Methods). While the future climate response in near-surface temperature is maximum in winter (DJF, see Fig. 1b), the seasonal forcing simulations suggest that the forcing season causing the strongest annual temperature response is spring (Fig. 2), and to a somewhat lesser degree summer. Interestingly, the spring and summer forcing combined contribute about 40% to the total wintertime temperature response. Even more surprising, summer forcing causes a much larger response in autumn and winter than in summer itself. In contrast, winter forcing is important for the wintertime response, but hardly for other seasons. The winter response is thus to a large degree governed by non-winter forcing, mainly through storage/release of ocean energy and associated feedbacks and possibly through changing atmospheric circulation. This clearly demonstrates that the near-surface Arctic temperature response to any climate forcing depends greatly on the season in which the forcing occurs, with the spring season being most effective. The only viable mechanism to invoke a surface air temperature response in seasons other than the forcing season relates to ocean storage and release of energy, which in turn is strongly modulated by sea ice (thickness) changes^5^.

**Figure 3:**
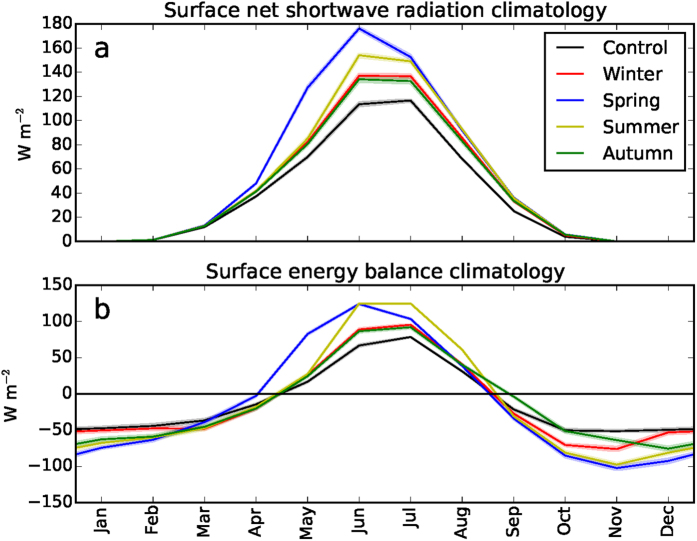
Simulated monthly Arctic mean surface energy budget components for each of the simulations (see Methods). (**a**) Net shortwave radiation, (**b**) Net surface flux (i.e. radiative fluxes plus turbulent fluxes). Downward fluxes are defined positive. The legend shows the forcing season. The uncertainty band represents the 95% confidence interval of the mean.

The concurrent response in Arctic sea ice cover clearly peaks for spring and summer forcing (Fig. 2). Spring forcing causes maximum sea ice decline in all other seasons, including the winter (even more than for winter forcing itself). This is due to the ice-albedo feedback amplifying the sea-ice retreat over the summer months^23^: the extra energy in spring thins the sea ice and/or creates melt ponds, lowering the surface albedo and allowing spring and summer insolation to more effectively warm the surface (Fig. 3a) and melt away sea ice^24^. In fact, reflected solar radiation in the Arctic peaks in spring, when both sea ice cover and insolation are relatively high, meaning that any change in spring sea ice has a profound effect on absorbed solar radiation. The ice-albedo feedback thus strongly amplifies the response only if the forcing occurs in the season in which the seasonal ice-albedo feedback is mainly active (spring/summer) and the additional energy is used to melt ice. This leads to enhanced absorption of shortwave radiation of the surface (Fig. 3a), increased sea surface temperatures, earlier onset of melt and an associated decline in sea ice cover. This amplified response for spring forcing is effectively carried over to subsequent seasons by storage of heat in the open Arctic Ocean, likely amplified by water vapour and cloud feedbacks^25^, leading to delayed freeze-up and thinner sea ice. The autumn sea ice response is indeed most pronounced for spring radiative forcing, which corresponds very well with observations-based correlations between enhanced incoming longwave radiation in spring and reduced autumn sea ice extent^23^.

Since sea ice effectively regulates the ocean-atmosphere energy exchange by acting as a lid, the total air-sea flux changes also exhibit a pronounced seasonal cycle (Fig. 2, Fig. 3b). In summer, nearly all additional longwave forcing is used to either melt sea ice or warm the upper Arctic ocean; this energy thus hardly contributes to warming of the lower atmosphere in this season, but it is instead stored and subsequently released in autumn/winter (Fig. 3b). A considerable part of the upward energy flux used to warm the lower atmosphere during autumn/winter originates from spring/summer forcing (Fig. 2). In contrast, a large portion of the additional energy is immediately returned to warm the lower atmosphere (and is partly lost to space) in case of autumn and winter surface forcing. Additionally, the limited impact of winter forcing on the response in other seasons can be attributed to the negative feedback between ice growth and ice thickness, meaning that ice thickness anomalies following a winter forcing will effectively decay in subsequent seasons. An extra downward forcing is thus most effective when the climatological net surface forcing is already downward (in late spring and summer, see Fig. 3b), so that the additional energy is predominantly used to warm the ocean and melt sea ice. In the winter months, the energy from the relatively mild and ice-depleted ocean surface is more easily radiated upward and thereby warms the overlying atmosphere (Fig. 3b). The stable stratification of the wintertime Arctic boundary layer reinforces warming of the near-surface atmosphere.

The seasonally varying response as shown in (Fig. 2) is purely the result of internal climate mechanisms since the magnitude of the applied radiative forcing was similar in all seasons. However, anthropogenic greenhouse forcing in the Arctic exhibits a pronounced seasonal cycle, peaking in late spring and summer^22^, which can be attributed mainly to the vertical distributions of temperature, water vapour and clouds, as well as to the temperature dependence of the emission/absorption characteristics of greenhouse gases. Intrusions of relatively warm and humid air from lower latitudes also lead to positive anomalies in moisture content and cloud amount, which especially in spring cause a considerable longwave forcing at the surface that contributes to enhanced sea ice melt later in the year^23^. Moreover, internal climate feedbacks, for instance those related to sea ice retreat and the associated enhanced surface evaporation and cloud formation, may lead to climate forcings that exhibit strong seasonal variations. In any case, the climate radiative forcing tends to peak in the seasons during which the Arctic system is most sensitive to additional forcing. With spring sensitivity being about 50% higher than in winter (Fig. 2), the impact of enhanced spring/summer forcing on the annual temperature response reinforces the response from internal climate mechanisms alone, increasing the seasonality of the temperature response (maximum in winter) as well as the magnitude of the annual response. The seasonality of Arctic surface radiative forcing thus exerts a comparatively strong impact on annual mean Arctic warming (compared to the hypothetical situation in which the forcing were seasonally invariant).

**Figure 4:**
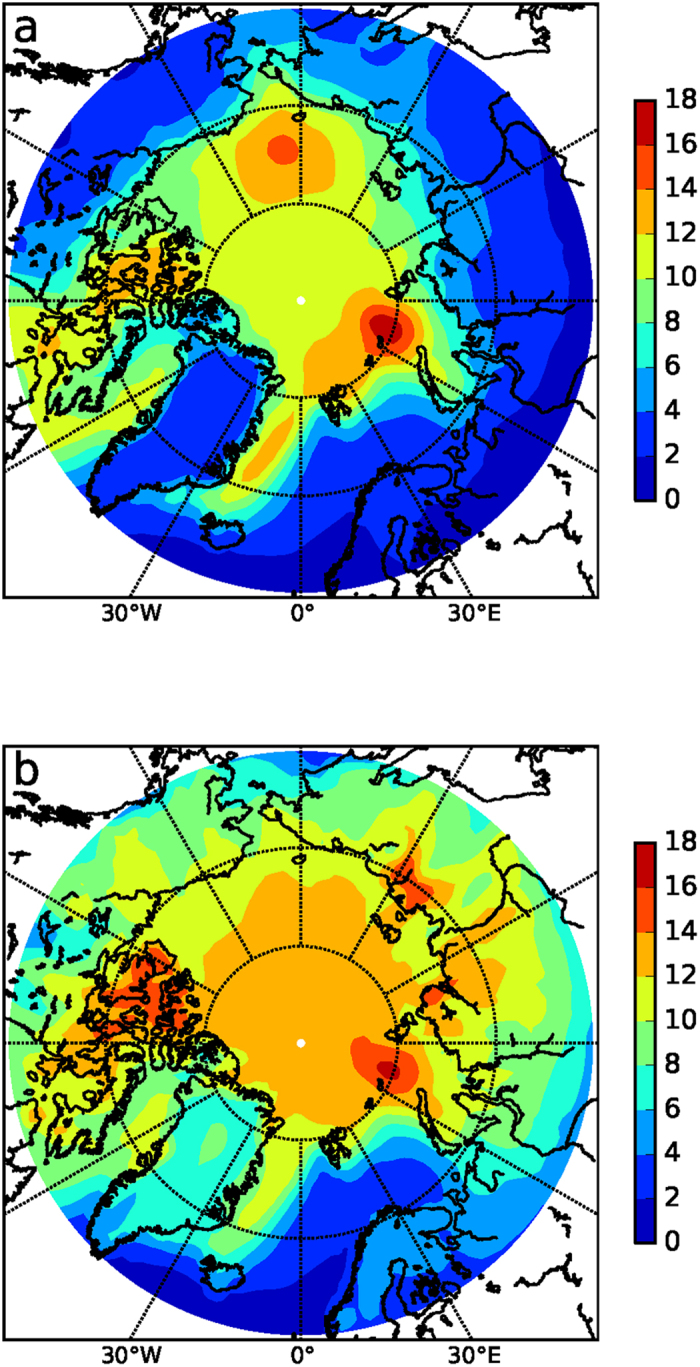
Simulated winter (DJF) temperature response as a function of seasonal radiative forcing (see Methods). (**a**) Response for spring (MAM) forcing, (**b**) Response for winter (DJF) forcing. The maps were made using Python V2.7 (www.python.org).

Over the Arctic Ocean, the ‘transfer’ of energy from one season to the other through storage and release of energy (Fig. 3b) is obviously most effective in the sea-ice retreat regions, since these are the locations where a strong increase in upward energy flux can occur, heating the lower atmosphere in autumn/winter. Therefore, the effect of spring forcing on the surface temperature response in winter peaks in the sea ice retreat regions (the periphery of the Arctic region), especially in the Barents Sea (Fig. 4a). Remarkably, in these regions the winter temperature response due to spring forcing is even larger than that due to winter forcing itself. In contrast, winter forcing causes a somewhat more uniform response (Fig. 4b) centered over the Arctic. Being effectively decoupled from the Arctic Ocean by sea ice, the direct winter forcing is mainly used to warm the (low thermal inertia) stably stratified lower atmosphere. Also, winter forcing yields a much larger surface air temperature response over the subarctic continents compared to spring forcing. Spring/summer forcing evidently exhibits a different geographical imprint compared to winter forcing in terms of the wintertime temperature response (and thereby the annual response, since this is governed by winter warming).

## Discussion

Because Arctic radiative forcing peaks in spring/summer, it contributes strongly to Arctic winter and annual warming, especially in the peripheral sea ice regions where, according to our climate model simulations, spring forcing even dominates winter warming through seasonal storage and release of energy in the Arctic Ocean. To understand Arctic warming and its huge seasonal cycle, as well as related processes (such as sea ice decline and precipitation increases^14^) it is therefore imperative to study not only the governing feedbacks but also assess the impact of seasonally-varying radiative forcing. This also implies that an accurate representation of changes in the seasonality of radiative forcing (which are dominated by clouds^11^, see Methods) is crucial to correctly project both the magnitude and the pattern of future Arctic warming. Uncertainties in Arctic radiative fluxes (which peak in summer^11^) mainly affect Arctic winter and annual warming^10^, meaning that not only uncertain Arctic feedbacks but also the (spring and summer) radiative forcing contribute to the intermodel spread in Arctic climate change^13^. Current changes in the Arctic climate peak in Autumn (in particular November). With ongoing Arctic warming and further reductions in sea ice, the changes will probably become more winter centered, with peak warming shifting towards the winter (see Fig. 1b), similar to the response simulated here (which represent an ‘artificial’ future warming relative to the present-day state).

Ongoing sea-ice decline is expected to spark human activities in the Arctic (e.g., shipping, fishery, mining, tourism), which will lead to increased emissions of radiatively-active constituents^15^,^26^, such as soot^27^, especially in summer (also soot originating from summertime sub-Arctic fires will enter the Arctic^27^). By lowering the sea-ice albedo^15^, the additional soot may induce a net radiative surface forcing during summer. The seasonal timing of anthopogenic radiative forcing (in whatever form) is most powerful when it occurs in seasons when the surface is climatologically gaining energy (spring and summer), so future anthropogenic emissions peaking in summer may reinforce Arctic (winter) warming and sea ice retreat.

## Methods

We used the global climate model EC-Earth V2.3^9^ (one of the CMIP5 models) to assess the mechanisms relating seasonally-varying radiative forcing to Arctic warming and sea ice retreat. EC-Earth V2.3 includes the following components: atmosphere, ECMWF’s Integrated Forecast System (IFS cycle 31r1), resolution T159L62, including HTESSEL as land surface module; ocean, NEMO V2, resolution 1 deg.; sea ice, LIM2, resolution 1 deg; all coupled through the OASIS3 coupler^9^. The performance of EC-Earth in terms of its simulation of the present-day climate is satisfactory, even though parts of the (sub) Arctic have too high winter temperatures, especially the continental regions of Siberia and Canada, while the central Arctic is somewhat too cold. We carried out 44-year simulations for perpetual year 2006 climate forcing (the model is in equilibrium for year 2006 radiative forcing), in which we added an artificial (or ‘ghost’) additional downward longwave radiative forcing of 30 W m^−2^ to the surface of the entire Arctic region north of 70 °N (we chose a relatively strong radiative forcing to obtain a climate response larger than the interannual variability yet small enough to avoid possible nonlinear effects becoming dominant; we additionally carried out simulations with smaller/larger forcing, and concluded that the first-order average Arctic response roughly linearly depends on the magnitude of the forcing); the forcing was applied in each season separately over the entire length of the simulation (hence each year). Thus, in total we performed 5 simulations: 4 simulations with additional forcing in winter (December-January-February, DJF), spring (March-April-May, MAM), summer (June-July-August, JJA) and autumn (September-October-November, SON), respectively, and a control simulation without additional forcing. Note that the seasonal forcing of 30 W m^−2^ translates to an annual forcing of 7.5 W m^−2^, comparable to the RCP8.5 radiative forcing (but applied here only to the Arctic). The seasonal longwave forcing of 30 W m^−2^ corresponds to 15, 10, 13 and 17% of the modelled climatological (of the control run) downward longwave radiation in DJF, MAM, JJA and SON, respectively. The results in Figs 2, 3 and 4 represent the differences between the additional forcing simulations and the control simulation over the final 30 years of the simulations (over which the residual trend is small). In this way the contributions of the feedbacks (for seasonally constant forcing) and of the (seasonally varying) radiative forcing on the seasonal climate response can effectively be separated. Obviously, the applied ghost forcing is a simplified representation of real radiative forcings, but previous studies^22^,^28^ have shown that this method nonetheless provides important insights into the primary response of the climate system. Note that the 30-year average climate state representing the year 2006 forcing (the control simulation) is used as a climatology to compare our sensitivity simulations to.

A possible caveat in our simulations is the absence of a transition zone in which the 30 Wm^−2^ forcing gradually reduces to zero, both spatial (at 70°N) and temporal (at the beginning and ending of seasons), rather than instantaneous. Even though such a ‘soft’ transition (instead of the ‘hard’ one used here) probably affects the details of the response (e.g. atmospheric dynamics), it is unlikely to have a considerable effect on the overall (i.e. Arctic mean) temperature response. Hence, we do not expect the first-order results to be sensitive to minor changes at the edges. Our ‘hard transition’ forcing has the advantage that results are easy to interpret, moreover this simple forcing is also easy to apply (in other studies), and therefore facilitates intercomparison of results.

One of the most uncertain issues in terms of a longwave radiation response concerns the role of clouds. Among CMIP5 models, Arctic cloud cover varies significantly. Also, most CMIP5 models exhibit biases in especially Arctic cloud phase, with excessive cloud ice and insufficient cloud water content^11^,^29^. This leads to an overestimate of surface cooling in winter and spring, too strong surface inversions and biases in TOA radiative fluxes. EC-Earth V2.3 has a single prognostic variable for cloud condensate mass with a temperature-driven partitioning between cloud liquid and solid mass, and also generally underestimates cloud liquid mass in the Arctic. This deficiency may affect the climate response resulting from enhanced radiative forcing.

## Additional Information

**How to cite this article**: Bintanja, R. and Krikken, F. Magnitude and pattern of Arctic warming governed by the seasonality of radiative forcing. *Sci. Rep.* **6**, 38287; doi: 10.1038/srep38287 (2016).

**Publisher's note:** Springer Nature remains neutral with regard to jurisdictional claims in published maps and institutional affiliations.
